# Exploratory behavior is associated with the cognitive speed in male chestnut thrushes

**DOI:** 10.1093/cz/zoad055

**Published:** 2023-12-05

**Authors:** Yingqiang Lou, Yuqi Zou, Yun Fang, Yuehua Sun

**Affiliations:** Key Laboratory of Animal Ecology and Conservation Biology, Institute of Zoology, Chinese Academy of Sciences, 1 Beichen West Road, Chaoyang District, Beijing 100101, China; Department of Biology, University of Konstanz, Konstanz 78464, Germany; Key Laboratory of Animal Ecology and Conservation Biology, Institute of Zoology, Chinese Academy of Sciences, 1 Beichen West Road, Chaoyang District, Beijing 100101, China; Key Laboratory of Animal Ecology and Conservation Biology, Institute of Zoology, Chinese Academy of Sciences, 1 Beichen West Road, Chaoyang District, Beijing 100101, China

**Keywords:** animal personality, animal cognition, learning ability, spatial memory test, speed-accuracy trade-off

## Abstract

Intra-individual variation in cognitive abilities has been widely reported in animals. Recent studies have found that individual cognitive performance varies with personality traits in a wide range of animal taxa, with a speed-accuracy trade-off between cognition and personality traits. Few studies investigated whether these relationships change depending on different contexts. Here we investigate whether the personality trait (as measured by exploratory behavior in a novel environment) is associated with cognition (novel skill learning and spatial memory) in wild male chestnut thrushes *Turdus rubrocanus*. Using an experimental novel skill-learning task set-up, we found that fast-exploring individuals explored the experimental device (a cardboard with 8 opaque cups) sooner than slow-exploring individuals. Exploratory behavior was not associated with individual spatial memory performances or an individual’s capacity to learn the novel skill. Learning speed was positively associated with the difficulty of learning phases, and fast-exploring individuals used less trials to meet the learning criterion. In addition, fast-exploring individuals took less time to complete the 24-h spatial memory test, but the accuracy of the test was not significantly different between individuals who were more or less exploratory. We suggest that variation in personality traits associates with individual learning speed in cognitive tasks and that this relationship is context-dependent.

Cognition is defined as the acquisition, processing, storage, and use of information (Shettleworth 2009) and is associated with behaviors, such as foraging, dispersal, and mate choice (Shettleworth 2009; Cauchoix et al. 2016; Chen et al. 2019), thus affecting individual fitness (Cole and Quinn 2012). A growing number of studies have found that individual differences in cognition exists among animals (Gomes et al. 2020; Harris et al. 2020; Lucon-Xiccato et al. 2020; Chen et al. 2021). Moreover, it is becoming increasingly important to understand the factors which is associated with the variation in cognition (Harris et al. 2020), such as animal personality (Sih and Giudice 2012; Griffin et al. 2015; Gomes et al. 2020).

Animal personality is defined as animal’s consistent differences in behavior across time and contexts (Sih et al. 2004; Réale et al. 2007), and has been identified in many animal taxa (Dall et al. 2010; Réale and Montiglio 2021). In general, individuals could be categorized from proactive to reactive personality types (Sih et al. 2004): proactive individuals are more aggressive and active, exploring faster and bolder than more reactive individuals (Carere and Maestripieri 2013). Differences in personality traits tend to be associated with how individuals acquire information and respond to changing conditions (Sih and Giudice 2012; Udino et al. 2017), such as environmental and/or social conditions, and this may account for variation in cognition.

In recent years, an increasing number of studies have revealed that the relationships between animal personality and cognition (Carere and Locurto 2011; Dougherty and Guillette 2018), in taxa such as insects (Harris et al. 2020), fish (Lucon-Xiccato et al. 2019), birds (Gomes et al. 2020; Chen et al. 2021), and mammals (Johnson-Ulrich et al. 2018; Mazza et al. 2018). However, the results were highly variable across different conditions (Dougherty and Guillette 2018), even for the same species. For example, fast-exploring black-capped chickadees *Poecile atricapillus* learned faster in learning tasks (Guillette et al. 2009), whereas for other cognitive task, fast explorers did not perform better than slow explorers (Guillette et al. 2015). Furthermore, the difficulty of cognitive tasks could also affect the relationship between personality and cognition (Titulaer et al. 2012; Chang et al. 2018); for example, experimental trials with jumping spider *Portia labiata* found that more aggressive individuals performed better in simple decision-making tasks, while docile individuals did better with more difficult tasks (Chang et al. 2018).

Sih and Giudice (2012) proposed that proactive individuals often make inaccurate, but fast decisions in cognitive tasks, while reactive individuals are slower but more accurate. Current empirical evidence for this theory is mixed. For example, fast and slow exploring birds are known to exhibit similar learning speed, but slow explorers have higher accuracy during test phases (Guillette et al. 2015). In mormyrid fish *Gnathonemus petersii*, bolder individuals make faster and more accurate decisions than shyer individuals (Kareklas et al. 2017). However, there is no nonlinear association between boldness and learning in Eastern water skink *Eulamprus quoyii*, with both bold and shy individuals performing better than intermediate individuals (Carazo et al. 2014).

In this study, we used a wild chestnut thrush *Turdus rubrocanus* population to investigate the relationships between exploratory behavior and cognition (novel skill learning and spatial memory). Exploratory behavior is a well-studied personality trait, having been found in many species (Baker et al. 2018; Dingemanse et al. 2020). In a recent study, we found that exploratory behavior was repeatable in this population (Lou et al. 2023), whereas another recent study on the same species found significant inter-individual variation in learning ability and spatial memory ability (Lou et al. 2022), whereby individuals could remember the location of food reward in 24 h spatial memory test (Lou et al. 2022). Thus, chestnut thrush represents a suitable species with which to study the relationship between exploratory behavior and cognition. The aims of this study are three-fold: (1) to investigate whether exploratory behavior is associated with the cognitive abilities (novel skill learning and spatial memory); (2) to determine whether the exploratory behavior is related to individual’s learning speed in novel skill-learning task; and (3) to determine whether there is a relationship between exploratory behavior and speed-accuracy trade-offs in the spatial memory test. To test these issues, we conducted novel skill-learning tasks and spatial memory tests to estimate individual cognitive abilities.

## Materials and Methods

### Study site and subjects

The study was performed at Badu village in Lianhuashan Nature Reserve, Gansu Province in China (34.67°N,103.50°E), from October to November in 2019 and April to August in 2020. The study area is located on the edge of forests and dominated by agricultural land (Sun et al. 2003). The chestnut thrush is a widespread species in southwestern China (Zheng 2017), with the breeding period occurring from late April to late August in our study area (Zhao and Sun 2018). We used mist nets to catch chestnut thrushes before and after breeding seasons in 2019 and 2020. We distinguished the adults and juveniles based on bill color (Lou et al. 2022): the bill color of juveniles is black or mixed black and yellow in the year that they hatch, and the bill color of adults is yellow.

### Measurement of exploratory behavior

To measure the exploratory behavior, we conducted an open field test at the field station in 2019 and 2020 shortly after capturing the birds (less than 20 min). We tested each bird separately between 0700 and 1800 in a sealed room (4.0 m × 2.4 m × 2.3 m) with 5 artificial trees and artificial light. Before conducting the exploratory tests, we acclimated each bird by placing it in a small cage (25 cm × 25 cm × 25 cm) connected to the test room, and darkened the cage for 10 min. After this acclimatization period, the door was opened, allowing the birds to enter the test room, which was illuminated. Movement behaviors (hop, fly, and walk) of each individual during the first 2 min in the test room were measured, and the cumulative number of each individuals’ movement was recorded as the exploratory score (Dingemanse et al. 2002). Exploratory score was positively associated with the visited area (*r =* 0.807, *P <* 0.001, *N =* 116). Consequently, individuals with higher exploratory scores were regarded as faster explorers. In 2019 and 2020, we measured the exploratory behavior 223 times in 169 adult birds (87 males and 82 females) during the breeding seasons (2 individuals were measured three times and 31 individuals were measured twice), and 43 times in 43 juveniles (37 males and 6 females) during the non-breeding season. To calculate the repeatability of exploratory behavior, we tried to catch the bird twice. The intervals between measurements ranged from 7 to 42 days (mean *N =* 17 days).

### Individuals housing

After conducting measurements of exploratory behavior, the chestnut thrushes were housed in 35 × 35 × 70 cm cages on a 11:13 h light: dark regime in the field station, which replicated natural conditions (Lou et al. 2022). We kept all individuals in visual, but not auditory, isolation from each other. Each cage contained a perch and 2 boxes (12 × 6 × 4.3 cm), one for food and one for water. Each bird could obtain 10 earthworms each hour during daytime (from 6:00 to 19:00), and 20 earthworms at 20:00.

### Novel skill-learning task

**Figure 1.**
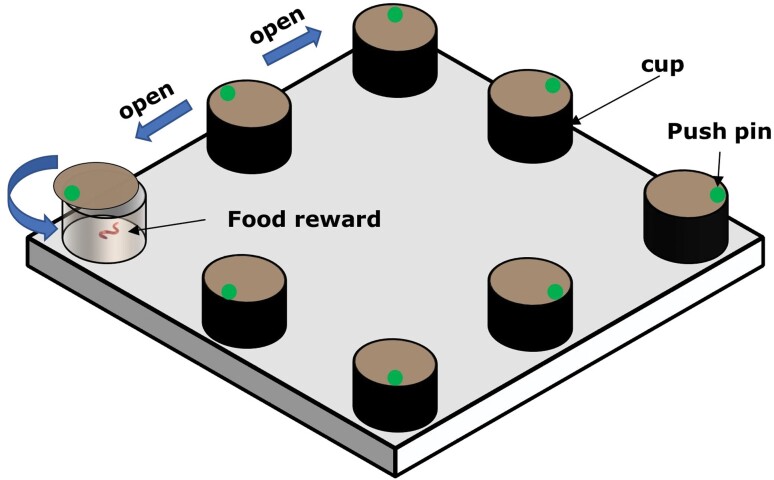
The apparatus of the novel skill-learning task. The apparatus consisted of 8 non-transparent cups, each was covered by a swivel lid that was secured with a small push pin (green dot).

In both 2019 and 2020, we conducted a novel skill-learning task on chestnut thrushes on each bird in a 35 × 35 × 70 cm cage without perches or boxes. Following the same methods described by Lou et al. (2022), each bird was trained to learn how to open the swivel lids. In the task, we used a device which included 8 opaque cups (3 cm deep, 5 cm diameter) that were covered with black insulating tape and fastened to a cardboard (34 × 34 cm) (Figure 1). The novel skill-learning task included 4 phases. For the first phase, all the cups were open, without any covering lids. Freshly dead earthworms (*Pheretima* sp.) were put in 3 random cups as food rewards, and these locations of the cups remained the same for each individual learning trial. After a bird entered the apparatus, it was free to search the open cups. The time taken by each thrush to begin to search the apparatus in the first trial was recorded as a measurement of approach latency. For the second phase, half of each cup was covered by a circle swivel lid with a push pin, whilst for the third phase, 75% of each cup was covered with a lid. The final fourth phase was considered to be the most difficult phase, involving covering each cup entirely with the lids, thus requiring individuals to peck the lid open to obtain the food rewards. Birds were defined as passing a phase when they had found and eaten at least one food reward in the 3 consecutive trials. If individuals failed to obtain the food reward in 3 consecutive trials, we placed them back into the previous phase, which they needed to complete again in order to progress to the next phase. We recorded the number of trials that a thrush used to pass each phase as learning speed of each phase. If individuals used their tails or wings to open the lids (deliberately or unexpectedly), we closed the lids again, in order for the trial to be repeated.

Each individual had 3 min time period to search and obtain food rewards in each trial. After each trial, we spent at least 1 min checking all cups and refilling any with new food rewards. If individuals did not reach the learning criterion in phase 3 or 4 after 25 trials, we would stop the task and categorized these individuals as non-learners. Our previous study showed that all individuals still had a strong motivation to forage after the tasks, suggesting that their performance to learn the novel skill was not affected by satiety (see Lou et al. 2022). In all experiments, the experimental apparatus was accessible to the birds only during the test trials and the individuals were also visually isolated to avoid potential effects of copying or social learning. All learning trials of each individual were finished in a single day, and these processes were observed and recorded by a micro-camera (WNK L6).

### Spatial memory test

We conducted spatial memory tests one hour after the completion of the novel skill-learning task. Each test used the same apparatus and was conducted only on individuals who had successfully learned to open lids (i.e., passed phase 4 of the novel skill-learning task). Before the test, we put earthworms into all cups to ensure that each cup had the olfactory scent of earthworms, which can efficiently prevent individuals from finding food reward by olfactory cues. To reduce the possibility of the birds remembering the locations of food rewards from the previous novel skill-learning task (which involved 3 cups), we randomly selected one of the remaining 5 cups to be the spatial memory cup (food reward) for each individual. This test was composed of 3 phases: the first phase allowed the birds to search all the cups with completely covered to locate the food reward cup. In the second phase, birds were given the opportunity to memorize the location of the food reward. During this phase, individuals were tasked with a 5-min search for the food reward, repeated across 3 trials. Our recent study showed that individuals could remember the locations in the second phase (Lou et al. 2022). The third phase tested the individual’s memory of the reward cup 24 h after passing the second phase, and it was performed only once. The number of cups searched before opening the food reward cup in the third phase was recorded as the spatial memory score, with a lower spatial memory score indicating higher accuracy (Ashton et al. 2018). Accuracy was calculated as the ratio of the number of searched cups until the opening the food reward cup, and it was calculated as 1/(spatial memory score + 1). Individuals were considered to have performed perfectly when accuracy was 1 and worst when accuracy was 0.125. Phase 3 was stopped when the individuals found the food item or 1500s had passed. The time taken by each individual to complete the 24 h spatial memory test was also recorded. All trials were observed and recorded by a micro-camera (WNK L6).

### Data analysis

To calculate the repeatability of exploratory behavior in chestnut thrushes, we constructed linear mixed models (LMMs) with square-root transformed exploratory score as the response factor. To control for potential confounding factors, 7 fixed factors were included: year, Julian date, sex, test sequence of exploratory behavior (i.e., 1: first, 2: second or 3: third test), test time (where 1200 = 0, 1300 = 1, 1100 = −1, etc.), context (i.e., 1: before breeding, 2: incubation, 3: nestling period or 4: after breeding) and age (adult and juvenile). Individual identity (bird ID) was used as a random variable. All continuous variables were mean centered and standardized. The “rptGaussian” function in R was used to calculate the repeatability (R 2015). We used the above LMMs of exploratory behavior to run 1000 simulations using the *arm* package (Gelman et al. 2022). The averaged best linear unbiased predictors (BLUPs) for the intercepts of each individual were used as the exploratory score in the following analyses (Dingemanse et al. 2020), since this method is unbiased although less precise (Dingemanse et al. 2020). Following Nakagawa and Schielzeth (2010), we also retained the individuals with only one measure in the analysis. For simplicity, we termed the average BLUPs of the exploratory score as the exploratory score. We measured the exploratory behavior of 208 chestnut thrushes in 2019 and 2020, including 169 adults (87 males and 82 females) and 39 juveniles (33 males and 6 females), and exploratory behavior was repeatable across years and time (*R =* 0.578, *P <* 0.001). A total of 52 males (18 adults and 34 juveniles) were measured for both cognitive abilities and exploratory behavior, and these data were used for subsequent analysis.

We used the Spearman correlation to examine the relationship between exploratory score and approach latency in the novel skill-learning task. A generalized linear mixed model with a binomial distribution was used to investigate the relationship between whether individuals passed the novel skill-learning task and exploratory behavior, with approach latency, age, and test time as fixed factors. We used another LMM to investigate whether the learning phase, age, exploratory score, approach latency, test time, year, and the interaction between exploratory score and learning phase affect the learning speed of each learning phase. Three further LMMs were used to investigate whether exploratory score, approach latency, test time, and Julian date were associated with the spatial memory score, the accuracy, and time spending in the 24 h spatial memory test. Bird ID was regarded as a random factor in all models. To reduce the potential collinearity of fixed factors, we checked the Variable Inflation Factor. We used a multi-model inference approach, based on Akaike’s information criterion corrected for small sample sizes (AICc) to select the final models (Burnham and Anderson 2004) using the function “dredge” in the MuMIn package (Barton 2016). We generated average models from the models within 2 AICc by using the function “model.avg” (Burnham and Anderson 2004). The AIC values for the top 8 candidate models of each model were preseanted as Supplementary Material (Supplementary Table S1). All statistical analyses were conducted in R version 3.5.1 (R 2015). The descriptive statistics in the text are mean ± standard deviation (SD).

## Results

A total of 52 chestnut thrushes participated in a novel skill-learning task, with a mean approach latency of 5.17 ± 6.41 min, ranging from 0.05 min to 25 min. Approach latency was significantly associated with chestnut thrushes’ exploratory score (*r =* −0.280, *P =* 0.044, *N =* 52): faster explorers began to search the apparatus significantly earlier than slower explorers.

**Figure 2.**
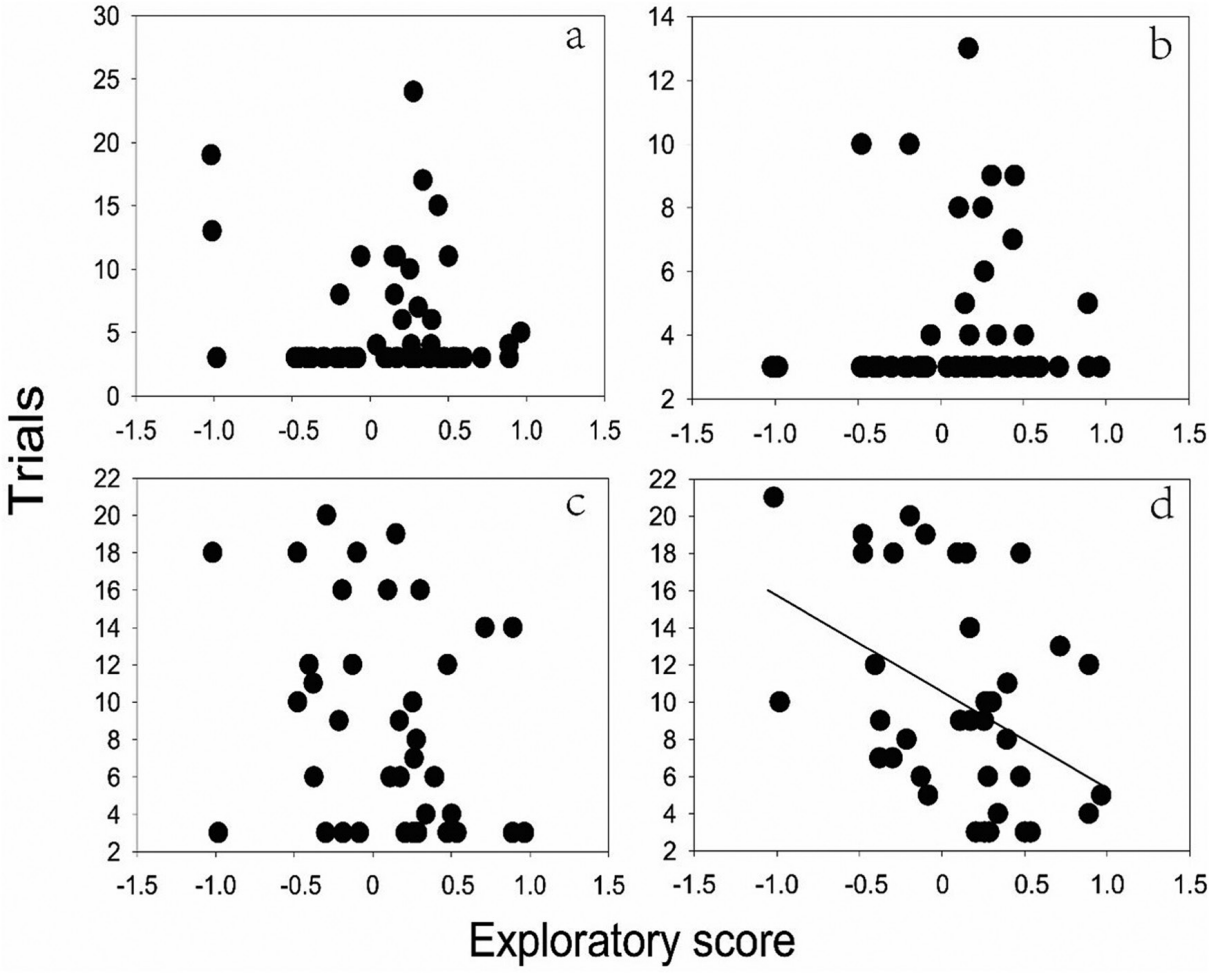
The relationship between the exploratory score and trials in different learning phases of novel skill-learning task in male chestnut thrushes. The learning phases (A–D) increased in difficulty, with phase “a” being the easiest and phase “d” being the most difficult. Each circle represents an individual. (A) first leaning phase, all the cups were open; (B) second learning phase, half of each cup was covered by a lid; (C) third learning phase, 75% of each cup was covered by a lid; (D) fourth learning phase, 100% of each cup was covered by a lid. The line was the significant line.

A total of 37 chestnut thrushes successfully learned the novel skill-learning task while 15 individuals failed. Whether they could learn the novel skill was not related to any factors in our study (age: Estimate = 0.515, CI = [−0.847, 1.872], Supplementary Table S1). Furthermore, the learning speeds of learning phase 1 to learning phase 4 were 5.65 ± 4.79, 4.17 ± 2.37, 8.94 ± 5.67 and 10.22 ± 5.73, respectively. Learning speed was associated with learning phases: individuals undertaking the fourth learning phase required more trials than the other phases (Table 2). Learning speed was also determined by the interaction between exploratory score and learning phase (Table 1). For each separate learning phase, we found that fast explorers learned the novel skill faster than slow explorers in the fourth learning phase (*t =* −2.754, *P =* 0.009, *N =* 37; Figure 2D), but not in other 3 phases (phase 1: *t =* −1.045, *P =* 0.487, *N =* 52; phase 2: *t =* 0.247, *P =* 0.741, *N =* 52; phase 3: *t =* −3.411, *P =* 0.090, *N =* 38; Figure 2A–C). There was no effect of other factors tested on the learning speed (Table 1).

**Table 1:** The linear mixed model revealed the effects of predictors on the learning speed in male chestnut thrushes

Predictors	Estimate	SE	2.5%	97.5%
ES	1.401	1.813	−2.178	4.979
LP	1.978	0.308	**1.340**	**2.556**
ES × LP	−1.577	0.660	−**2.880**	−**0.274**
Age	1.058	0.917	−0.753	2.868

Bold indicated the significant factor.

ES, exploratory score; LP, learning phase.

**Table 2:** The linear mixed model revealed the effects of predictors on time spending on 24 h spatial memory test in male chestnut thrushes

Predictors	Estimate	SE	*P*
ES	−284.897	127.867	**0.026**
ET	−22.390	11.579	0.053
TT	−7.264	24.796	0.770
Age	−139.873	154.406	0.365

Bold indicated the significant factor.

ES, exploratory score; ET, exploratory tendency; TT, test time.

In total, 37 individuals participated in the 24 h spatial memory test and 11 individuals failed to find the location. Of the 37 participants, 26 individuals spent 320.61 s ± 299.10 s to find the food location (ranging from 35 s to 1041 s), with the number of cups searched in the spatial memory test ranging from 0 to 6 (18/26 of individuals located the reward cup through non-random sampling, mean ± SE = 3.12 ± 2.32). Of the 26 individuals who found the location of food reward, we found that their spatial memory score was not associated with exploratory score (Estimate = −0.517, CI = [−2.609, 1.575]) or age (Estimate = −1.021, CI = [−3.146, 1.104]). Fast explorers spent less time to find the food reward cups than slow explorers in the 24 h spatial memory test (Table 2). Furthermore, the accuracy was not associated with any factors (Supplementary Table S1), as the best model only included the intercept and random factor (Supplementary Table S1).

## Discussion

In this study, we investigated the relationship between individual differences in exploratory behavior and cognitive ability (novel skill learning and spatial memory performance) in male chestnut thrushes. We found that both adult and juvenile chestnut thrushes exhibited consistent behavioral differences in exploratory behavior across time. The latency of fast explorers to approach the apparatus was shorter than that of slower explorers. Exploratory behavior was not associated with whether individuals could learn novel skill or spatial memory score, and it could predict the speed in novel skill-learning task and actual time taken by individuals to finish in spatial memory test. Importantly, we found no evidence to support the personality and cognitive speed-accuracy trade-off hypothesis.

Exploratory behavior was a significant predictor of approach latency in novel skill-learning tasks, with fast explorers beginning to search the apparatus (which was unfamiliar to all individuals) more quickly than slow explorers. Sih et al. (2004) suggested that exploratory behavior covaries with other personality traits, such as neophobia and boldness, therefore comprising of a suite of correlated behavioral traits, that is, a “behavioral syndrome,” with fast explorer animals typically less reluctant to approach novel objects (Réale et al. 2007; Biondi et al. 2020). Thus, fast explorers may require less time to adapt to novel apparatus and initiate searching more quickly during novel skill-learning tasks.

We found no relationship between exploratory behavior and whether chestnut thrushes could learn a novel skill, which contrasts with other studies (Guillette et al. 2009; Medina-Garcia et al. 2017; Dougherty and Guillette 2018). In our study, all experimental chestnut thrushes were wild-caught, and they may have formed positive or negative feedback loops from previous life experiences (Sih et al. 2015). For example, fast explorers obtained higher benefits (or higher costs) by learning novel skill in the past, which may make them more prone (or unwilling) to participate in our experiments. Thus, the relationship between exploratory behavior and whether individuals could learn novel skill may be associated with many previous life-experience factors, and further studies on these relationships are needed.

We found that for wild chestnut thrushes, the relationship between exploratory behavior and learning speed was context-dependent, and a negative relationship was only found in the most difficult phase (phase 4) of the novel skill-leaning task. Similar results have also been found in other studies (Coleman et al. 2005; Titulaer et al. 2012; Chang et al. 2018). The process of learning involves forming associations between cues and rewards, which is closely linked to attention (Dukas 2004). Fast explorers may exhibit a heightened level of attention in cognitive tasks (Griffin et al. 2015), allowing them to form associations between novel skill and food rewards more quickly during the learning process (Guillette et al. 2015; Dougherty and Guillette 2018). On the other hand, slow explorers may be prone to ignoring certain cues and thus require more trials before meeting the learning criterion. In our study, the first 3 learning phases were considered to be relatively easier than phase 4, as all individuals were able to see the food and obtain rewards by pecking the lids. However, in the final phase, the cups were covered by lids and the individuals had to rely on their prior learning experience to access the food rewards. Observations during the fourth phrase showed some fast-exploring chestnut thrushes spent more time touching and trying to open the lids, while slow individuals would stop after a few failing unsuccessful attempts, which resulted in more learning trials. Therefore, personality-dependent variation in learning speed may be affected by the differences in individual attention, especially in the most difficult learning phase.

The results of our study revealed no significant relationship between spatial memory score and exploratory behavior, which is consistent with previous findings in other bird species (Guillette et al. 2009; Medina-Garcia et al. 2017). Sih and Giudice (2012) suggest that the relationship between exploratory behavior and spatial cognition may be affected by an individual’s ability to collect and accumulate information. Fast explorers may rely on a few salient landmarks to remember their surroundings, while slow explorers may use multiple landmarks and cues to build a more detailed mental map. In our study, the size of the apparatus may have been relatively small with limited cues, but this may not be a limitation for chestnut thrushes as they have the ability to store spatial memory over long distances. Unpublished field observations from our study site reveal that chestnut thrushes are fairly mobile, traveling nearly 200 m on a daily basis between different foraging areas, which offers ample opportunity for individuals to collect and accumulate spatial information. Thus, the relatively small size of the apparatus used in our study may have resulted in similar spatial performance among both fast and slow explorers.

We found that fast explorers obtained the food rewards more quickly than slow explorers during the 24 h spatial memory test. Our experiments were conducted indoor, and had lower error cost, which allowed fast explorers to use trial-and-errors pecking to obtain food rewards quickly (Burns and Dyer 2008). Sih and Giudice (2012) propose that fast explorers may be inherently able to make rapid choices in cognitive tasks. Thus, the speed of decisions made by fast explorers may not be affected by the amount of information gathered, but rather by their ability to make decisions faster. Despite their faster in food acquisition, fast and slow explorers showed similar accuracy in the 24 h spatial memory test. Given that the chestnut thrush is not a food-caching species, this suggests that both fast and slow explorers may have similar spatial cognition abilities. Furthermore, the apparatus used in spatial memory test may have been relatively easy for both fast- and slow-exploring chestnut thrushes to gather sufficient information to remember the locations of food rewards. Further studies are needed to increase the distance between locations to test the spatial cognition ability in chestnut thrushes.

In conclusion, we found that exploratory behavior could not predict the cognitive performance in chestnut thrushes, and the relationship between exploratory behavior and learning speed is context-dependent. Our results did not support the personality and cognitive speed-accuracy trade-off hypothesis in the spatial memory task. Taken together, our work added to the growing evidence on the relationship between personality and cognition, and further studies are needed to explore which factors could affect the relationship.

## Supplementary Material

Supplementary material can be found at https://academic.oup.com/cz.

zoad055_suppl_Supplementary_Tables_S1
