# Supplementary material for: Exploratory behavior is associated with the cognitive speed in male chestnut thrushes
Source: Curr Zool. 2023 Dec 5;70(6):707–13. doi: 10.1093/cz/zoad055 (PMC11634674; doi:10.1093/cz/zoad055)
Supplement: zoad055_suppl_Supplementary_Tables_S1 [file zoad055_suppl_supplementary_tables_s1.docx]

Table S1. The top 8 candidate models explaining variation in novel skill learning performance, learning speed and spatial memory performance of chestnut thrushes, as determined via the Akaike’s Information Criterion.

| Cognitive ability | Candidate Model | AICc | ΔAICc | Weights |
| --- | --- | --- | --- | --- |
| Learning performance | Null model | 66.725 | 0 | 0.298 |
|  | Age | 68.376 | 1.651 | 0.131 |
|  | TT | 68.832 | 2.107 | 0.104 |
|  | ES | 68.939 | 2.214 | 0.099 |
|  | ET | 68.980 | 2.255 | 0.097 |
|  | TT + Age | 70.513 | 3.788 | 0.045 |
|  | Age + ES | 70.725 | 4.000 | 0.040 |
|  | ET + Age | 70.727 | 4.002 | 0.040 |
| Learning speed | LP + Age + ES + LP* ES | 1064.567 | 0 | 0.312 |
|  | LP + LP* ES + ES | 1065.393 | 0.827 | 0.206 |
|  | ET + ES + LP + LP* ES + Age | 1066.782 | 2.215 | 0.103 |
|  | ET + ES + LP + LP* ES | 1067.143 | 2.576 | 0.086 |
|  | TT + ES + LP + LP* ES + Age | 1067.264 | 2.697 | 0.081 |
|  | TT + ES + LP + LP* ES | 1068.162 | 3.595 | 0.052 |
|  | ES + LP + Age | 1069.108 | 4.541 | 0.032 |
|  | ET + ES + LP + LP* ES + Age + TT | 1069.482 | 4.915 | 0.027 |
| Spatial memory score in the 24 h spatial memory test | Age | 123.219 | 0 | 0.251 |
|  | Null model | 123.392 | 0.173 | 0.230 |
|  | ES | 123.966 | 0.747 | 0.173 |
|  | Age + ES | 124.316 | 1.097 | 0.145 |
|  | TT | 126.577 | 3.358 | 0.047 |
|  | TT + Age | 127.192 | 3.973 | 0.034 |
|  | ES + TT | 127.491 | 4.272 | 0.030 |
|  | Age + ET | 128.367 | 5.148 | 0.019 |
| Time taken to complete in the 24 h spatial memory test | ES + Age + ET + TT | 331.878 | 0 | 0.862 |
|  | ES + ET + Age | 336.403 | 4.525 | 0.090 |
|  | ES + Age + TT | 338.395 | 6.517 | 0.003 |
|  | ES + ET + TT | 340.814 | 8.936 | 0.001 |
|  | ES + Age | 343.529 | 11.651 | 0.001 |
|  | Age + ET + TT | 344.262 | 12.384 | 0.001 |
|  | ES + ET | 346.319 | 14.441 | 0.001 |
|  | ES + TT | 347.427 | 15.549 | 0.001 |
| Accuracy in the 24 h spatial memory test | Null model | 9.699 | 0 | 0.845 |
|  | ES | 14.730 | 5.031 | 0.068 |
|  | Age | 15.230 | 5.531 | 0.053 |
|  | TT | 17.375 | 7.676 | 0.018 |
|  | ET | 19.433 | 9.734 | 0.001 |
|  | ES + Age | 20.230 | 10.531 | 0.001 |
|  | TT + Age | 22.074 | 12.375 | 0.001 |
|  | ES + TT | 22.753 | 13.054 | 0.001 |

ES: exploratory score; ET: exploratory tendency; TT: test time; LP: learning phase.
